# Effective Removal of Calcium and Magnesium Ions from Water by a Novel Alginate–Citrate Composite Aerogel

**DOI:** 10.3390/gels7030125

**Published:** 2021-08-25

**Authors:** Zhuqing Wang, Zhongmin Feng, Leilei Yang, Min Wang

**Affiliations:** College of Chemistry and Chemical Engineering, Anqing Normal University, Anqing 246133, China; wangzhq@aqnu.edu.cn (Z.W.); anqingnu@gmail.com (Z.F.); leileiyang0118@gmail.com (L.Y.)

**Keywords:** sodium citrate, aerogel, adsorbent, sodium alginate, calcium ion, magnesium ion

## Abstract

In this work, a novel alginate/citrate composite aerogel (CA–SC) was synthesized by chemical grafting technology combined with vacuum freeze-drying method, and CA–SC was used for removing calcium (Ca^2+^) and magnesium (Mg^2+^) ions from water. The experimental results indicate that the as-prepared CA–SC has a high affinity for Ca^2+^ and Mg^2+^ and can remove 96.5% of Ca^2+^ (or 96.8% of Mg^2+^) from the corresponding solution. The maximum adsorption capacities of CA–SC for Ca^2+^ and Mg^2+^ are 62.38 and 36.23 mg/g, respectively. These values are higher than those of the most reported Ca^2+^-sorbents and Mg^2+^-sorbents. The CA–SC adsorbent can be regenerated through a simple pickling step, and its adsorption performance keeps stable after repeated use. Analysis of the adsorption mechanism shows that the CA–SC combines Ca^2+^ and Mg^2+^ in water mainly through coordination effect.

## 1. Introduction

Although Ca^2+^ and Mg^2+^ in water have no direct health hazards, they have unwanted effects, such as scaling on water appliances and reduced washing efficiency of soap and detergent [1]. Prolonged consumption of drinking water containing Ca^2+^ and Mg^2+^ increases the incidence of stone in the human filtration system. In industry, the precipitation of calcium and magnesium salts forms scale, hinders heat conduction, and even causes a boiler explosion in severe cases. Therefore, developing a new method of removing excessive Ca^2+^ and Mg^2+^ from water has important application prospects [2]. At present, the methods for Ca^2+^ and Mg^2+^ removal from water mainly include precipitation [3], boiling [4], use of lime–soda ash [5], ion exchange [6], electrodialysis [7], and adsorption [8,9]. Among such treatment methods, the adsorption method has attracted much attention in recent years because of its advantages of simple operation, recyclability, and low cost [10,11].

Recently, many nano-adsorbents have been developed to remove Ca^2+^ and Mg^2+^ from water. For example, El-Nahas et al. and Xue et al. utilized nano powder zeolite and mesoporous zeolite, respectively, for Ca^2+^ and Mg^2+^ removal in water [12,13]. Mahmoud et al. modified nano-silica particles using *Fusarium verticillioides fungus* to soften hard water [14]. Given the higher specific surface area and surface energy of these nano-adsorbents, they have higher adsorption capacities and adsorption rates for Ca^2+^ and Mg^2+^. However, nano-sized adsorbents usually require strict preparation conditions, such as high temperature, high pressure, and high-purity reagents [15]. The higher energy consumption and cost of raw materials limit the further large-scale application of these nano-adsorbents. In addition, recollecting the adsorbents from the solid–liquid mixture is difficult due to the small size of the nano-adsorbents.

Sodium citrate is a safe, non-toxic, water-soluble, biodegradable, and synthetic organic compound. It is often used as a flavoring agent and stabilizer in the food industry, and as an anticoagulant and a blood transfusion agent in the pharmaceutical industry [16,17]. In addition, sodium citrate is also a good chelating agent and has good complexing ability for metal ions, such as Ca^2+^ and Mg^2+^ in water. Alginate is a kind of natural polymer, which is often used in the treatment of heavy metal poisoning and as a drug sustained-release agent [18,19,20,21,22].

To effectively remove Ca^2+^ and Mg^2+^ ions in water, in this study, we used green sodium alginate and sodium citrate as raw materials to prepare a new low-cost, easy-to-recover calcium alginate–sodium citrate composite aerogel (named as CA–SC). In addition, we also characterized the morphology and chemical composition of the prepared adsorbent and evaluated its abilities in the treatment of water containing Ca^2+^ and Mg^2+^ ions.

## 2. Results and Discussion

### 2.1. Characterizations

**Figure 1:**
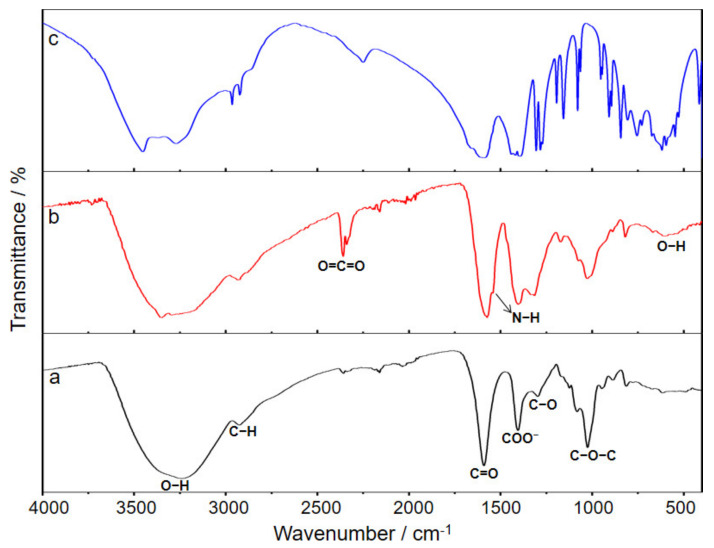
Infrared spectra of (**a**) sodium alginate, (**b**) CA–SC, and (**c**) sodium citrate.

The functional groups in the adsorbent were verified via spectroscopic characterization of sodium citrate, sodium alginate, and CA–SC. As shown in Figure 1, the broad absorption peak around 3240 cm^−1^ is an O–H stretching vibration, and the absorption peak around 2929 cm^−1^ belongs to the aliphatic C–H stretching vibrations [16]. The peak at 1590 and 1297 cm^−1^ can be assigned to C=O and C–O vibrations, respectively [23]. The absorption peak around 1020 cm^−1^ in sodium alginate and CA–SC can be assigned to C–O–C vibration. Compared with the infrared spectra of sodium alginate and sodium citrate, the new absorption peak at 1536 cm^−1^ in CA–SC is an N–H bond derived from the modified ethylenediamine [17]. In addition, compared with that of the raw material sodium alginate, the relative intensity of the –COO^−^ peak in CA–SC has increased somewhat. Although the –COO^−^ in alginate reacts with the –NH_2_ of ethylenediamine and consumes some –COO^−^ groups, the citrate modified on CA–SC has twice the –COO^−^ groups, which leads to an increase in –COO^−^ content in CA–SC [23].

**Figure 2:**
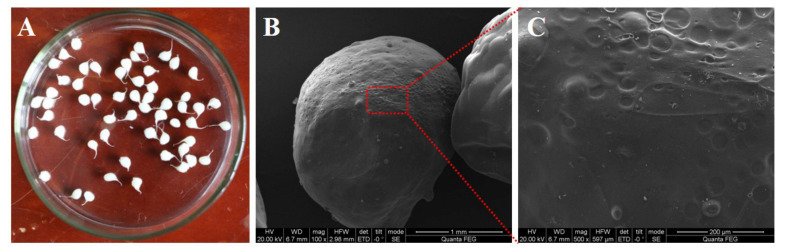
(**A**) Digital and (**B**,**C**) SEM photos of CA–SC; (**C**) is a partial enlargement of (**B**).

**Figure 3:**
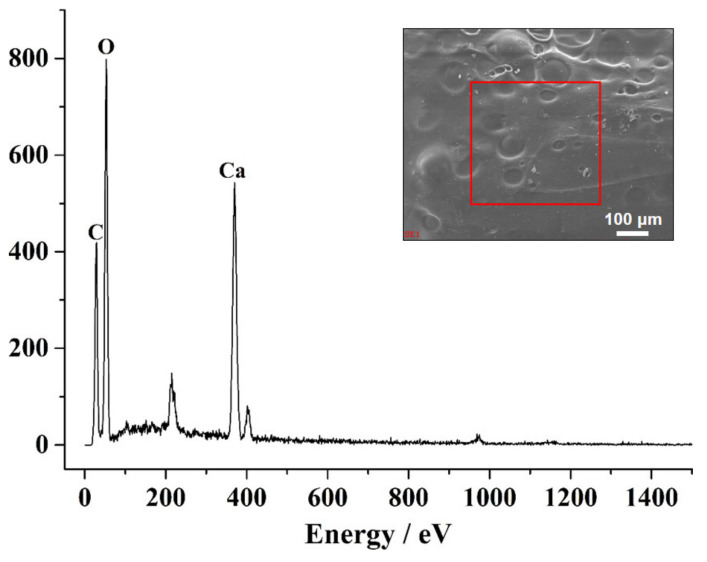
EDS energy spectrum of the partial surface of CA–SC. The inserted image is the selected surface of CA–SC.

Figure 2 shows that the synthesized CA–SC is a macroscopic, drop-like aerogel, and its macroscopic size improves its recyclability. The SEM images show that the CA–SC surface is uneven. Compared with a smooth surface, an uneven surface increases the contact area with the adsorbate, which promotes the increase in the adsorption capacity of the adsorbate. Further, CA–SC composition was analyzed using an energy dispersive X-ray spectrometer (EDS) (Figure 3). The EDS energy spectrum data show that CA–SC mainly contains carbon (C), oxygen (O), and calcium (Ca), and the mass percentage of C, O, and Ca of the CA–SC surface is 23.19%, 59.32%, and 17.49%, respectively. In addition, the CA–SC surface is rich in oxygen-containing functional groups.

### 2.2. Effect of the Concentrations of Sodium Alginate and Ethylenediamine

In the process of preparing CA–SC, we found that the concentration of sodium alginate and ethylenediamine had a great influence on the morphology of the final adsorbent. Therefore, different concentrations of sodium alginate and ethylenediamine were employed to prepare the CA–SC adsorbent. The results showed that as the concentration of sodium alginate increased, the shape of CA–SC obtained transformed from sheet to sphere. On another hand, when the reactant ethylenediamine remains greater, the color of CA–SC is darker. Therefore, 3.0 w/v% sodium alginate and 5.0 w/v% ethylenediamine were selected to prepare the CA–SC sorbent in this work.

### 2.3. Effect of Solution pH

**Figure 4:**
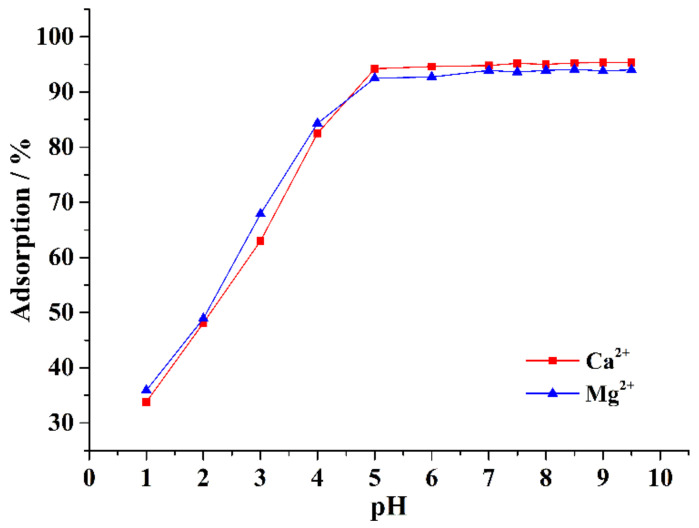
Influence of the acidity of the solution on the adsorption performance of CA–SC for Ca^2+^ and Mg^2+^.

Approximately 0.1 g of CA–SC was weighed, added into 50 mL of 1.5 mM Ca^2+^ (or Mg^2+^) solution of different pH (1, 2, 3, 4, 5, 6, 7, 7.5, 8, 8.5, 9, or 9.5), stirred for 6 h, and then filtered. The amount of remaining Ca^2+^ (or Mg^2+^) in the filtrate was determined by ICP-OES. It can be seen from Figure 4 that as the pH of the solution increases, the adsorption capacity of CA–SC for Ca^2+^ and Mg^2+^ gradually increases and becomes stable. When the solution pH value is less than 1.0, the removal efficiency of CA–SC for Ca^2+^ and Mg^2+^ is less than 35%. This result may be attributed to the protonation of the –COO^−^ and –NH groups on CA–SC under a strong acid environment, thereby reducing the amount of coordinated functional groups with Ca^2+^ and Mg^2+^. In addition, according to the solubility product rule, when the pH of a solution is higher than 9.78, Mg^2+^ in the solution begins to form Mg(OH)_2_ precipitation (when pH is higher than 12.78, Ca^2+^ begins to form Ca(OH)_2_ precipitation). Therefore, the optimal pH range of the solution is 5.0–9.5. Within this range, precipitation is absent, and only few protonated functional groups are present.

### 2.4. Influence of Contact Time

**Figure 5:**
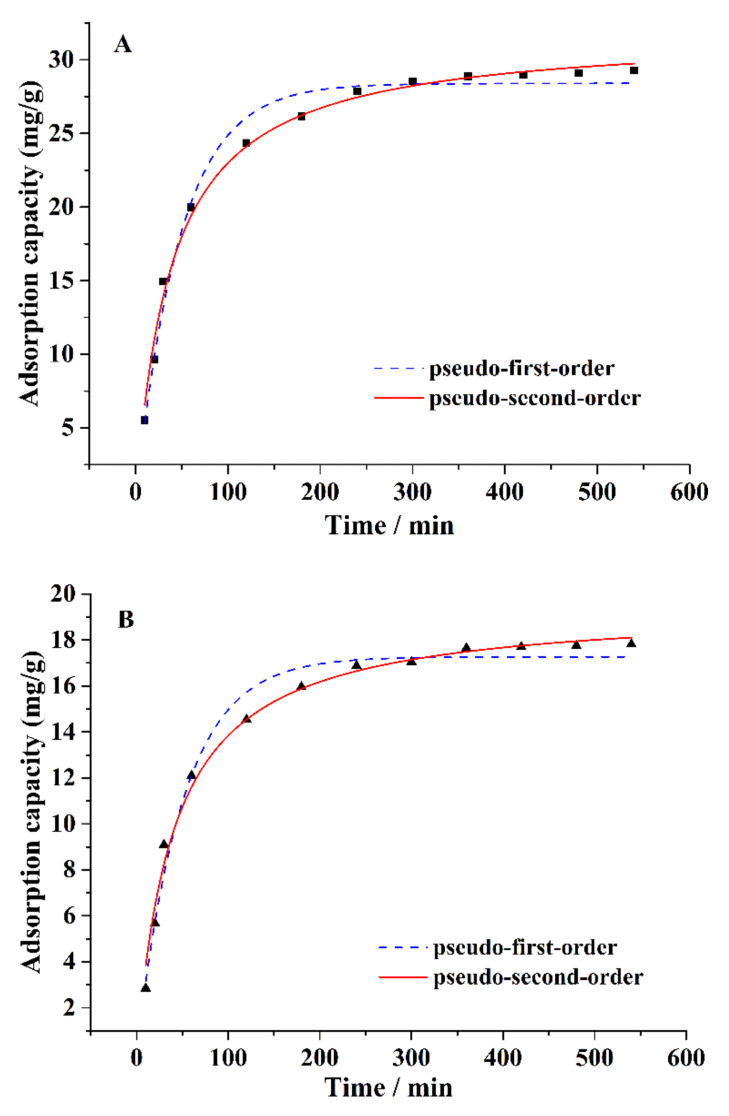
Adsorption kinetics of (**A**) Ca^2+^ and (**B**) Mg^2+^ on the CA–SC.

Approximately 0.1 g of CA–SC was weighed, added into 50 mL of 1.5 mM Ca^2+^ (or Mg^2+^), stirred for 10, 20, 30, 60, 120, 180, 240, 300, 360, 420, 480, or 540 min, and filtered. The amount of Ca^2+^ (or Mg^2+^) remaining in filtrate was measured using ICP-OES. As can be seen in Figure 5, as time increases, the adsorption capacity of CA–SC for Ca^2+^ or Mg^2+^ firstly increases and is kept stable. CA–SC can remove more than 80% of Ca^2+^ and Mg^2+^ in the corresponding solution with an adsorption time of 2 h, and reaches saturation with an adsorption time of 6 h. Therefore, the adsorption time selected in this study is 6 h.

In addition, we also used the pseudo-first-order and pseudo-second-order kinetic models to fit the experimental data. The derived kinetic models’ parameters for Ca^2+^ and Mg^2+^ onto CA–SC are given in Table 1. As indicated by the calculated R^2^ values, the pseudo-second-order kinetic equation is more consistent with the experimental data, indicating that chemical adsorption plays an important role in the adsorption process [24].

The pseudo-first-order kinetic equation is q_t_ = q_e_ (1 − exp(−k_1_t)), the pseudo-second-order kinetic equation is q_t_ = q_e_ (1 − 1/(1 + q_e_k_2_t)) [25,26], where k_1_ and k_2_ are the rate constant of pseudo-first-order and pseudo-second-order, respectively, q_t_ is the amount of metal adsorbed at a certain time (t), and q_e_ is the amount of metal adsorbed at equilibrium.

### 2.5. Influence of Ambient Temperature

**Figure 6:**
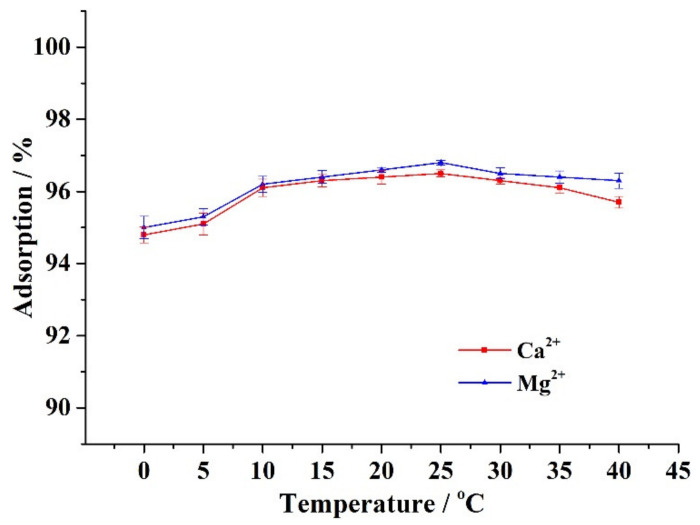
Influence of ambient temperature on the adsorption performance of CA–SC for Ca^2+^ and Mg^2+^.

Approximately 0.1 g of CA–SC was weighed, added into 50 mL of 1.5 mM Ca^2+^ (or Mg^2+^), stirred at 0 °C, 5 °C, 10 °C, 15 °C, 20 °C, 25 °C, 30 °C, 35°C, or 40 °C for 6 h, and filtered. Figure 6 shows that the ambient temperature has a minimal effect on the adsorption capacity of CA–SC. In the ambient temperature range of 0–40 °C, the removal efficiency of CA–SC for Ca^2+^ and Mg^2+^ remains at 94–97%, which indicates that CA–SC is suitable for treating water containing Ca^2+^ and Mg^2+^ at different temperatures.

### 2.6. Adsorption Ability of CA–SC

The adsorption abilities of CA–SC for different metal ions (Cu^2+^, Zn^2+^, Co^2+^, Pb^2+^, Cr^3+^, Ca^2+^, and Mg^2+^) are shown in Table 2. It can be seen from Table 2 that CA–SC has good affinity for Cu^2+^, Zn^2+^, Co^2+^, Pb^2+^, Cr^3+^, Ca^2+^, and Mg^2+^ and can remove more than 94% of the corresponding metal ions from the solution. This result may be attributed to the combination of carboxyl, hydroxyl, and amino functional groups on CA–SC with metal ions through coordination. In the presence of other ions (Cu^2+^, Zn^2+^, Co^2+^, Pb^2+^, and Cr^3+^), CA–SC can still remove 87.2% of Ca^2+^ and 86.5% of Mg^2+^ in the mixed solution. Thus, CA–SC can be used for treating wastewater containing heavy metal ions and can effectively soften hard water in the presence of multiple ions.

### 2.7. Maximum Adsorption Capacity

**Figure 7:**
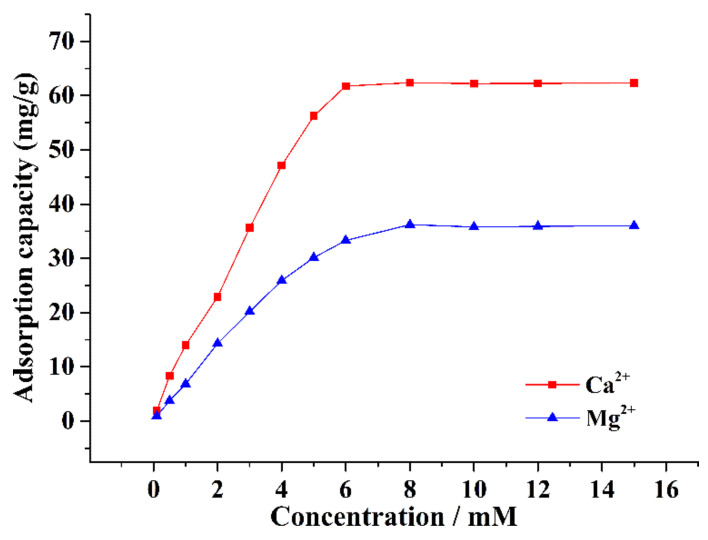
Maximum adsorption capacity of CA–SC for Ca^2+^ and Mg^2+^.

Approximately 0.1 g of CA–SC was weighed, added into 50 mL of different initial concentrations of Ca^2+^ or Mg^2+^ solutions (0.1, 0.5, 1.0, 1.5, 2.0, 3.0, 4.0, 5.0, 6.0, 7.0, 8.0, 10, or 15 mM), stirred at room temperature for 6 h, then filtered. The remaining Ca^2+^ and Mg^2+^ concentrations in the filtrate were determined by the ICP-OES. Figure 7 shows that with the increase in the initial concentration of Ca^2+^ and Mg^2+^, the adsorption capacity of CA–SC for Ca^2+^ and Mg^2+^ gradually increases and remains stable as the concentration further increases. When the initial concentration of Ca^2+^ and Mg^2+^ exceeds 8.0 mM, the adsorption capacity of CA–SC for Ca^2+^ and Mg^2+^ reaches saturation. Thus, the maximum adsorption capacities of CA–SC for Ca^2+^ and Mg^2+^ are 62.38 and 36.23 mg/g, respectively. These values are higher than those of the most reported Ca^2+^-sorbents and Mg^2+^-sorbents (Table 3).

### 2.8. Adsorption Mechanism

**Figure 8:**
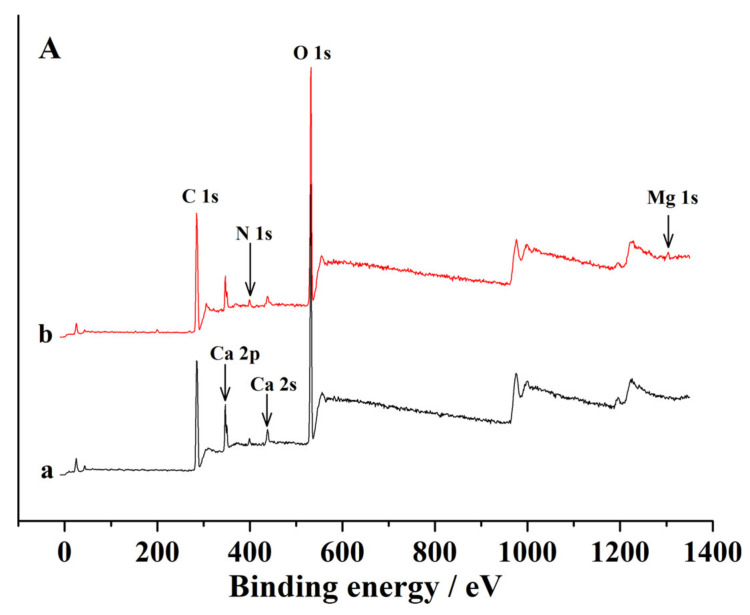

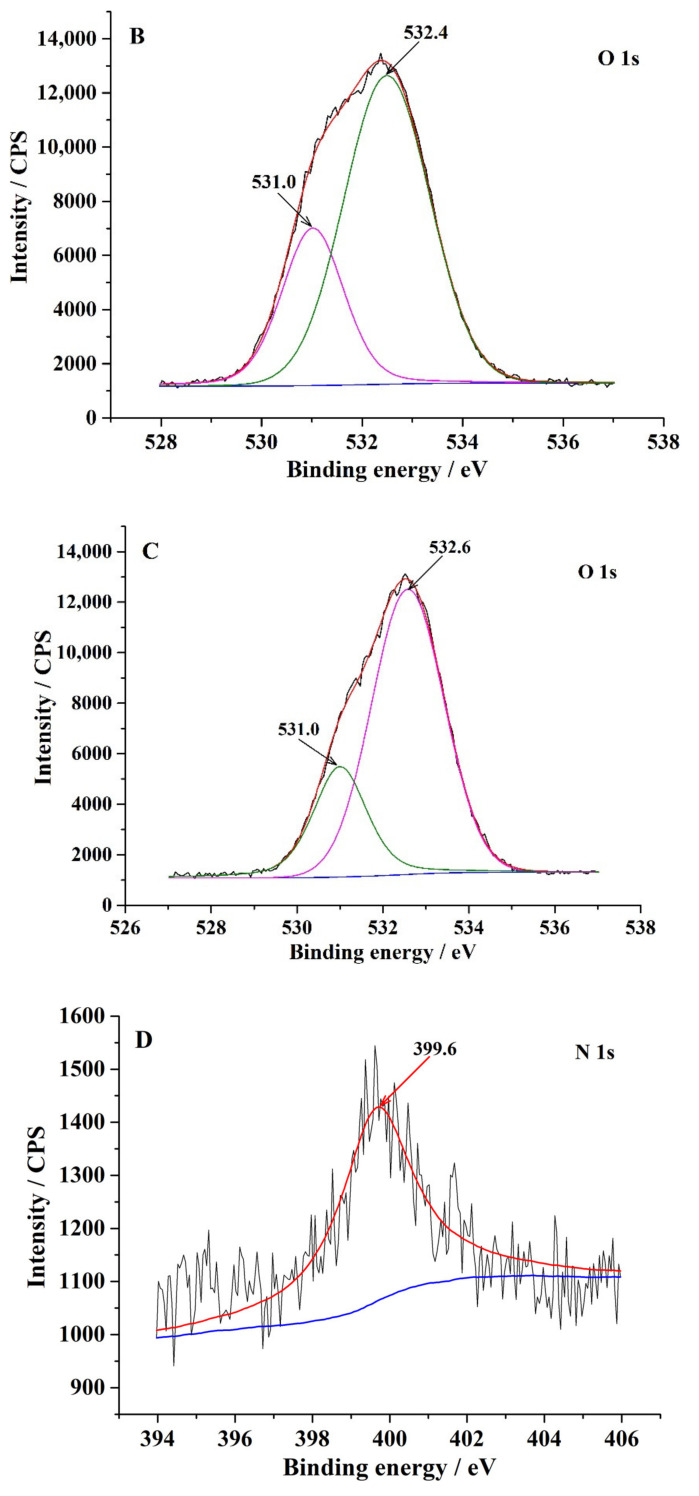

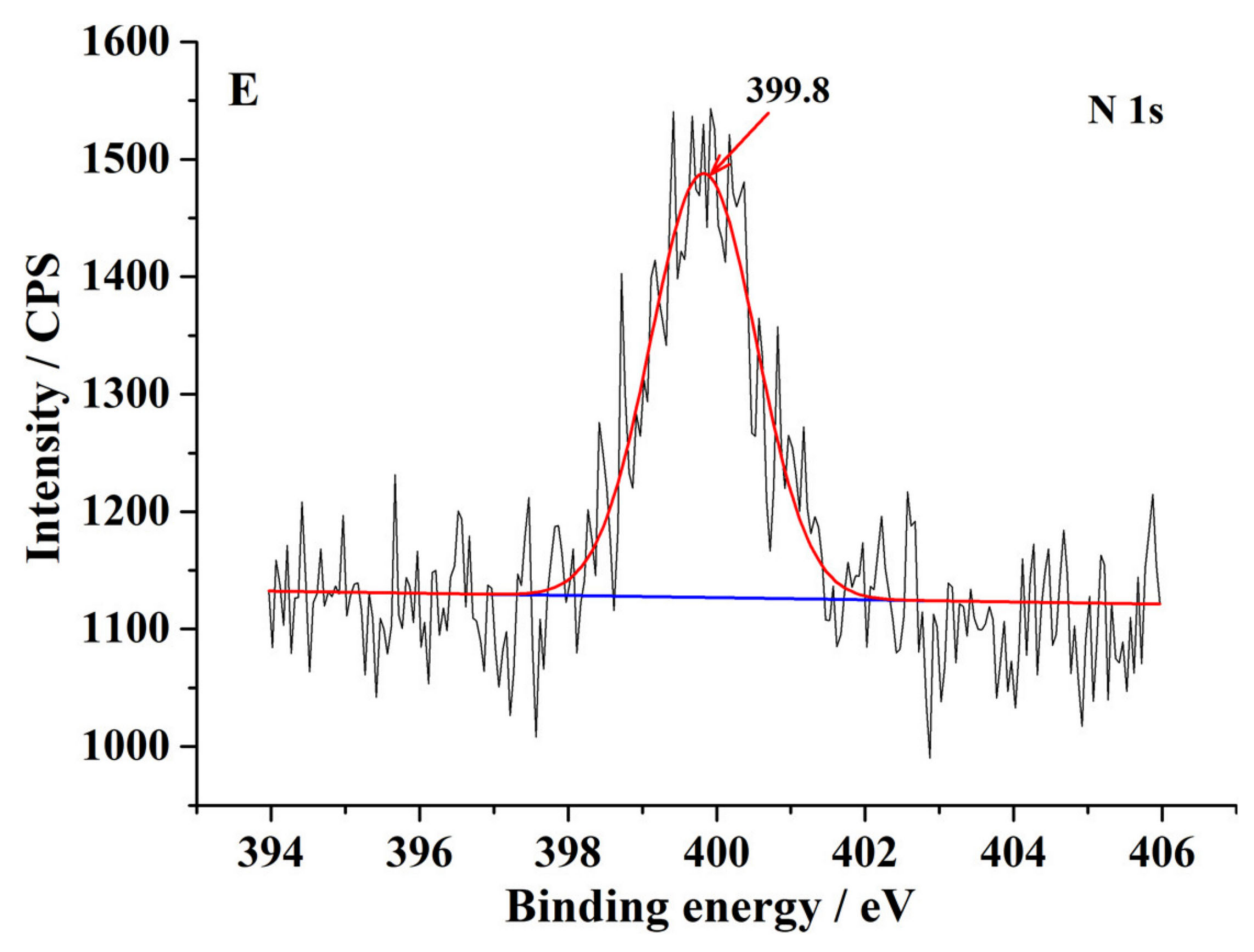
(**A**) XPS spectra of (**a**) CA–SC and (**b**) Mg^2+^-loaded CA–SC; O 1s of (**B**) CA–SC and (**C**) Mg^2+^-loaded CA–SC; N 1s of (**D**) CA–SC; and (**E**) Mg^2+^-loaded CA–SC.

**Figure 9:**
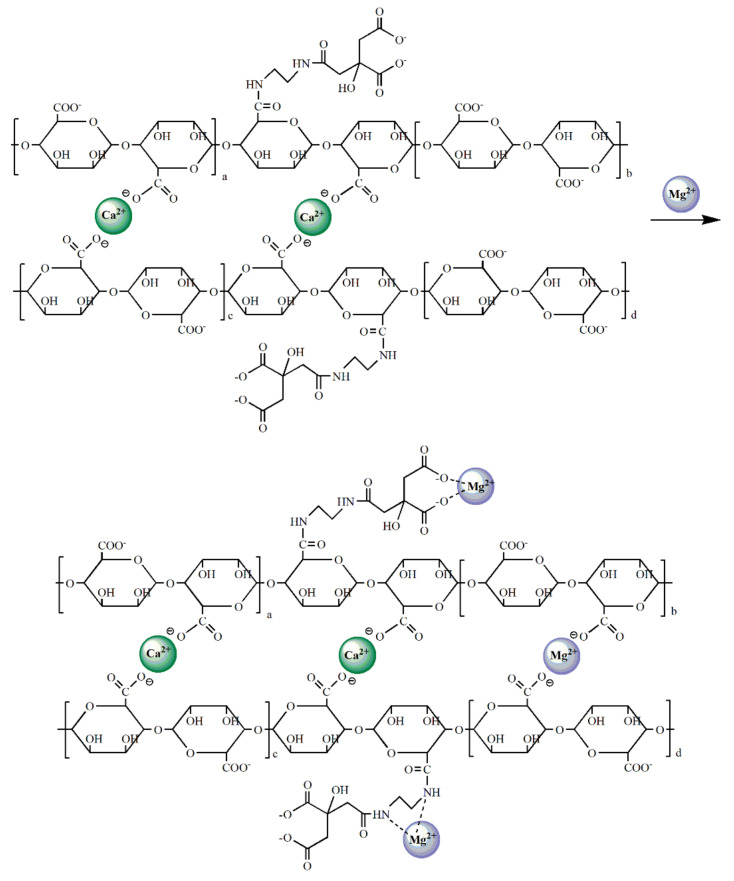
Possible adsorption mechanism of the CA–SC to Mg^2+^.

To clarify the mechanism of CA–SC binding with Ca^2+^ and Mg^2+^ ions, we performed XPS analysis on CA–SC samples before and after being loaded with Mg^2+^. As can be seen in Figure 8A, the peaks around 399.6, 437.8, and 532.4 eV can be assigned to N 1s, Ca 2s, and O 1s element peaks, respectively. The new Mg 2s peak at 1303.3 eV in (b) indicates that the CA–SC was successfully loaded with Mg^2+^ after immersion in a Mg^2+^-containing solution and is consistent with the ICP-OES measurement results (the change in Mg^2+^ concentration in the solution before and after adsorption). Further, we also analyzed the O 1s and N 1s spectra. As illustrated in Figure 8B–E, after loaded with Mg^2+^, the O 1s peak at 532.4 eV (C–O) and the N 1s peak at 399.6 eV of CA–SC shifted to 532.6 eV and 399.8 eV, respectively [36,37]. This may be caused by the lone pair electrons of N, O elements entering the empty sp^3^ hybrid orbitals of Mg^2+^ that reduced the electron cloud density of N, O atoms [16]. On the basis of the above analysis, we propose a possible adsorption mechanism for CA–SC to bind Mg^2+^ ions (Figure 9).

### 2.9. Regeneration and Recycling Performance

**Figure 10:**
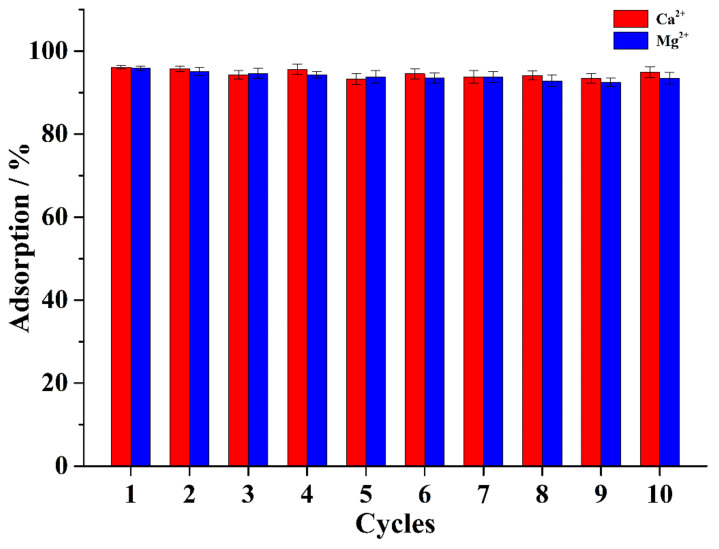
Adsorption/desorption cycle performance of the CA–SC.

In order to regenerate the adsorbent, nitric acid, acetic acid, hydrochloric acid, and EDTA were employed as the eluents to elute Ca^2+^ and Mg^2+^ ions on CA–SC. Experimental results show that 50 mM of nitric acid has the best elution effect, as it can elute more than 96.5% of Ca^2+^ (or Mg^2+^) ions on the adsorbent. Therefore, we chose 50 mM of nitric acid as the eluent to regenerate the adsorbent in this study. As shown in Figure 10, in 10 adsorption/desorption cycles, the removal efficiency of CA–SC for Ca^2+^ and Mg^2+^ ions were maintained between 93–97%. In addition, after nine cycles of use, the change in the CA–SC morphology was minimal. As such, CA–SC has good stability, and its physical and chemical properties remain stable after multiple cycles of use.

## 3. Conclusions

In summary, a novel CA–SC sorbent was successfully developed using green and safe sodium alginate and sodium citrate as raw materials. Through chemical coordination, the prepared CA–SC can effectively remove Ca^2+^ and Mg^2+^ in aqueous solutions. The maximum adsorption capacities for Ca^2+^ and Mg^2+^ reach 62.38 and 36.23 mg/g, respectively. In addition, CA–SC adsorbent can be regenerated by simple pickling, and its adsorption performance remains stable after repeated use. Thus, CA–SC has broad application prospects in the treatment of water containing Ca^2+^ and Mg^2+^ ions.

## 4. Materials and Methods

### 4.1. Reagents and Instruments

Sodium citrate, sodium alginate (average molecular weight: 270,000, 200 ± 20 mPa·s), 2-morpholineethanesulfonic acid, N-hydroxysuccinimide (NHS), N-(3-dimethylaminopropyl)-N′-ethylcarbodiimide (EDC), ethylenediamine, and metal salts were all purchased from J & K Scientific Ltd (Beijing, China). Except that sodium alginate is chemically pure, all other reagents used in this study were of analytical grade. The experimental water used in this work was double-distilled water. Lake water was collected from Anqing Linghu Lake, and river water was obtained from the Anqing section of the Yangtze River.

The macro and micro morphological images of CA–SC were taken using a Canon digital camera and an electronic scanning electron microscope (SEM), respectively. The chemical composition and functional groups of the adsorbent were determined using an infrared spectrometer and X-ray photoelectron spectrometer (XPS). The concentration of each metal ion in a solution was measured using an inductively coupled plasma emission spectrometer (ICP-OES).

### 4.2. Preparation of CA–SC

First, 1.5 g of sodium alginate was dissolved in 2-morpholineethanesulfonic acid buffer solution (pH 5.5), added with 0.08 g of EDC and 0.06 g of NHS successively, and mechanically stirred for 3 h. Then, 3.0 mL of ethylenediamine was added, and stirring was continued for 6 h. Sodium citrate (1.5 g) was added and stirring commenced for 6 h. The solution was added into the calcium ion solution by using a 5.0 mL syringe. The hydrogels were washed three times with distilled water and immersed in 50 mL of distilled water. They were placed in a vacuum freeze dryer, frozen, and vacuum dried to obtain the final CA–SC.

### 4.3. Adsorption and Desorption Experiments

For the adsorption experiment, about 0.1 g of CA–SC was weighed and added to 50 mL of 1.5 mM Ca^2+^ solution. The mixture was magnetically stirred for 6 h at room temperature, and then filtered. The remaining Ca^2+^ concentration in filtrate was determined using ICP-OES. The adsorption process of other metal ions is the same as above process, except that Ca^2+^ is replaced by target metal ion.

For the desorption experiment, Ca^2+^-loaded CA–SC was added to 50 mL of 50 mM nitric acid solution. The mixture was magnetically stirred for 3 h, filtered, and the sorbent was washed with distilled water. The amount of metal ions in the eluent was measured by the ICP-OES. The desorption process of Mg^2+^ is the same as above, except that Ca^2+^-loaded CA–SC is replaced by Mg^2+^-loaded CA–SC.

Adsorbent regeneration and circulation tests were performed by adding Ca^2+^-loaded CA–SC into 50 mL of 50 mM nitric acid solution. After magnetic stirring for 3 h, it was filtered, the sorbent was washed with distilled water, calcium hydroxide solution (20 mM), and distilled water in sequence, and dried in an oven at 50 °C for 4 h.

## Figures and Tables

**Table 1:** Kinetic parameters for Ca^2+^ and Mg^2+^ sorption onto the CA–SC.

Models	Formulas	Parameters	Ca^2+^	Mg^2+^
pseudo-first-order	q_t_ = q_e_ (1 − exp(−k_1_t))	q_e_ (mg/g)	28.395	17.266
		standard error for q_e_	0.395	0.268
		k_1_ (L/min)	0.021	0.020
		standard error for k_1_	0.001	0.001
		R^2^	0.984	0.982
pseudo-second-order	q_t_ = q_e_ (1 – 1/(1+q_e_k_2_t))	q_e_ (mg/g)	31.873	19.467
		standard error for q_e_	0.405	0.312
		k_2_ (L/min)	8.114 × 10^−4^	0.001
		standard error for k_2_	5.804 × 10^−5^	1.128× 10^−4^
		R^2^	0.994	0.991

**Table 2:** Adsorption ability of CA–SC for different metal ions.

CA–SC	Initial Concentration (mM)	Removal Efficiency (%)	Adsorption Capacity (mg/g)
In deionized water	1.5 (Ca^2+^)	96.5	29.0
	1.5 (Mg^2+^)	96.8	17.6
	1.5 (Cu^2+^)	96.6	46.0
	1.5 (Zn^2+^)	94.2	46.2
	1.5 (Co^2+^)	96.8	42.8
	1.5 (Cr^3+^)	96.8	37.8
	1.5 (Pb^2+^)	95.2	147.9
	1.5 (Ca^2+^, Mg^2+^, Cu^2+^, Zn^2+^, Co^2+^, Cr^3+^, Pb^2+^)	87.2 (Ca^2+^), 86.5 (Mg^2+^), 94.6 (Cu^2+^), 73.0 (Zn^2+^), 68.7 (Co^2+^), 87.3 (Cr^3+^), 88.9 (Pb^2+^)	26.2 (Ca^2+^), 15.8 (Mg^2+^), 45.1 (Cu^2+^), 35.8 (Zn^2+^), 30.3 (Co^2+^), 34.0 (Cr^3+^), 138.2 (Pb^2+^)
In tap water	1.5 (Ca^2+^)	92.9	27.9
	1.5 (Mg^2+^)	90.9	16.6
In lake water	1.5 (Ca^2+^)	91.2	27.4
	1.5 (Mg^2+^)	90.1	16.4
In river water	1.5 (Ca^2+^)	89.8	26.9
	1.5 (Mg^2+^)	88.8	16.2

**Table 3:** Adsorption capacities of the reported adsorbents for Ca^2+^ and Mg^2+^.

Adsorbent	Adsorbate	Maximum Adsorption Capacity (mg/g)	References
Mesoporous LTA zeolite	Ca^2+^, Mg^2+^	61.27 (Ca^2+^), 9.24 (Mg^2+^)	[13]
Modified bentonite	Ca^2+^, Mg^2+^	14.63 (Ca^2+^), 14.63 (Mg^2+^)	[27]
Alkaline modified pumice stones	Ca^2+^	57.2–62.3	[28]
Modified zeolite	Mg^2+^	26.2	[29]
Chemically modified cellulose	Ca^2+^, Mg^2+^	15.6 (Ca^2+^), 13.5 (Mg^2+^)	[30]
Sugar cane bagasse	Ca^2+^, Mg^2+^	46.1 (Ca^2+^), 23.5 (Mg^2+^)	[30]
Rice husk	Mg^2+^	3.87	[31]
Green tomato husk	Mg^2+^	6.76	[32]
Sugar cane bagasse modified with citric acid	Ca^2+^	26.52	[33]
Sugar cane bagasse modified with tartaric	Ca^2+^	14.72	[33]
Activated Chilean zeolite	Mg^2+^	0.774	[34]
Black carrot residues	Mg^2+^	3.871	[31]
Aluminosilicate chlorosodalite	Ca^2+^, Mg^2+^	49.26 (Ca^2+^), 32.25 (Mg^2+^)	[35]
CA–SC	Ca^2+^, Mg^2+^	62.38 (Ca^2+^), 36.23 (Mg^2+^)	This work
